# Supplementary material for: Embedded 3D Printing in Self‐Healing Annealable Composites for Precise Patterning of Functionally Mature Human Neural Constructs
Source: Adv Sci (Weinh). 2022 Jun 16;9(25):2201392. doi: 10.1002/advs.202201392 (PMC9443452; doi:10.1002/advs.202201392)
Supplement: Supplementary file 1 — Supporting information [file ADVS-9-2201392-s003.pdf]

ADVANCED SCIENCE
Open Access

Supporting Information

for *Adv. Sci.*, DOI 10.1002/advs.202201392

Embedded 3D Printing in Self-Healing Annealable Composites for Precise Patterning of Functionally Mature Human Neural Constructs

Janko Kajtez, Milan Finn Wesseler, Marcella Birtele, Farinaz Riyahi Khorasgani, Daniella Rylander Ottosson, Arto Heiskanen, Tom Kamperman, Jeroen Leijten, Alberto Martínez-Serrano, Niels B. Larsen, Thomas E. Angelini, Malin Parmar, Johan U. Lind and Jenny Emnéus**

Supplementary Materials for

Embedded 3D printing in self-healing annealable composites for precise patterning of functionally mature human neural constructs

Janko Kajtez, Milan Finn Wesseler, Marcella Birtele, Farinaz Riyahi Khorasgani, Daniella Rylander Ottosson, Arto Heiskanen, Tom Kamperman, Jeroen Leijten, Alberto Martínez-Serrano, Niels B. Larsen, Thomas E. Angelini, Malin Parmar, Johan U. Lind, Jenny Emnéus

This PDF file includes:

Figs. S1 to S5
Movies S1 to S3

Other Supplementary Materials for this manuscript include the following:
Movies S1 to S3

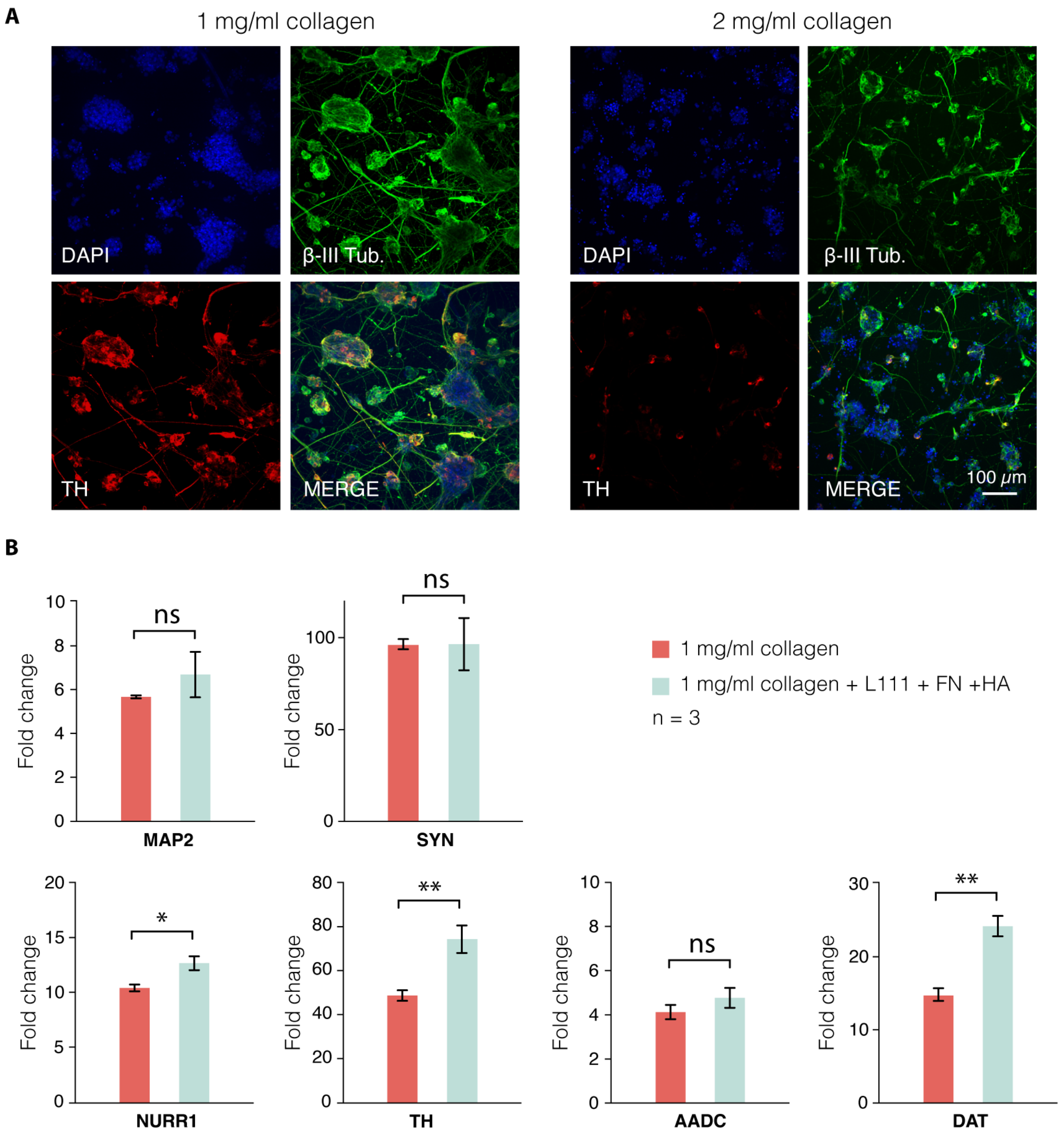

Fig. S1 Soft ECM-mimicking hydrogels promote hNSC differentiation into ventral midbrain neurons. (**A**) hNSCs were embedded in collagen hydrogels with varying collagen concentration (0.5 mg mL^{-1}, 1 mg mL^{-1}, 2 mg mL^{-1}). The lowest concentration did not crosslink sufficiently to provide 3D support to the cells. Immunocytochemistry after 4 weeks of differentiation indicates that softer hydrogels (1 mg mL^{-1}) provide a more permissive environment for expansion of cellular aggregates and growth and pathfinding of neuronal projections into the bulk of the hydrogel. Maximum intensity projections of fluorescence confocal images (150 µm thick optical slice) show that more extensive neuronal networks are formed in softer hydrogels marked by neuronal marker β-tubulin III, and higher expression of tyrosine hydroxylase (TH), a marker for dopaminergic neurons. (**B**) qRT-PCR gene expression analysis of hNSC differentiated for 4 weeks in collagen hydrogels ($2x10^6$ cells mL^{-1}, 1 mg mL-1 collagen type I) with or without addition of other ECM components (2 µg mL^{-1} laminin 111, 5 µg mL-1 fibronectin, 100 µg mL^{-1} hyaluronic acid). While no significant difference between the two conditions was observed in the expression of neural

markers, additional ECM components cause significant increase in expression of genes related to midbrain identity (NURR1, TH, DAT). Non-differentiated hNSCs were used as a reference.

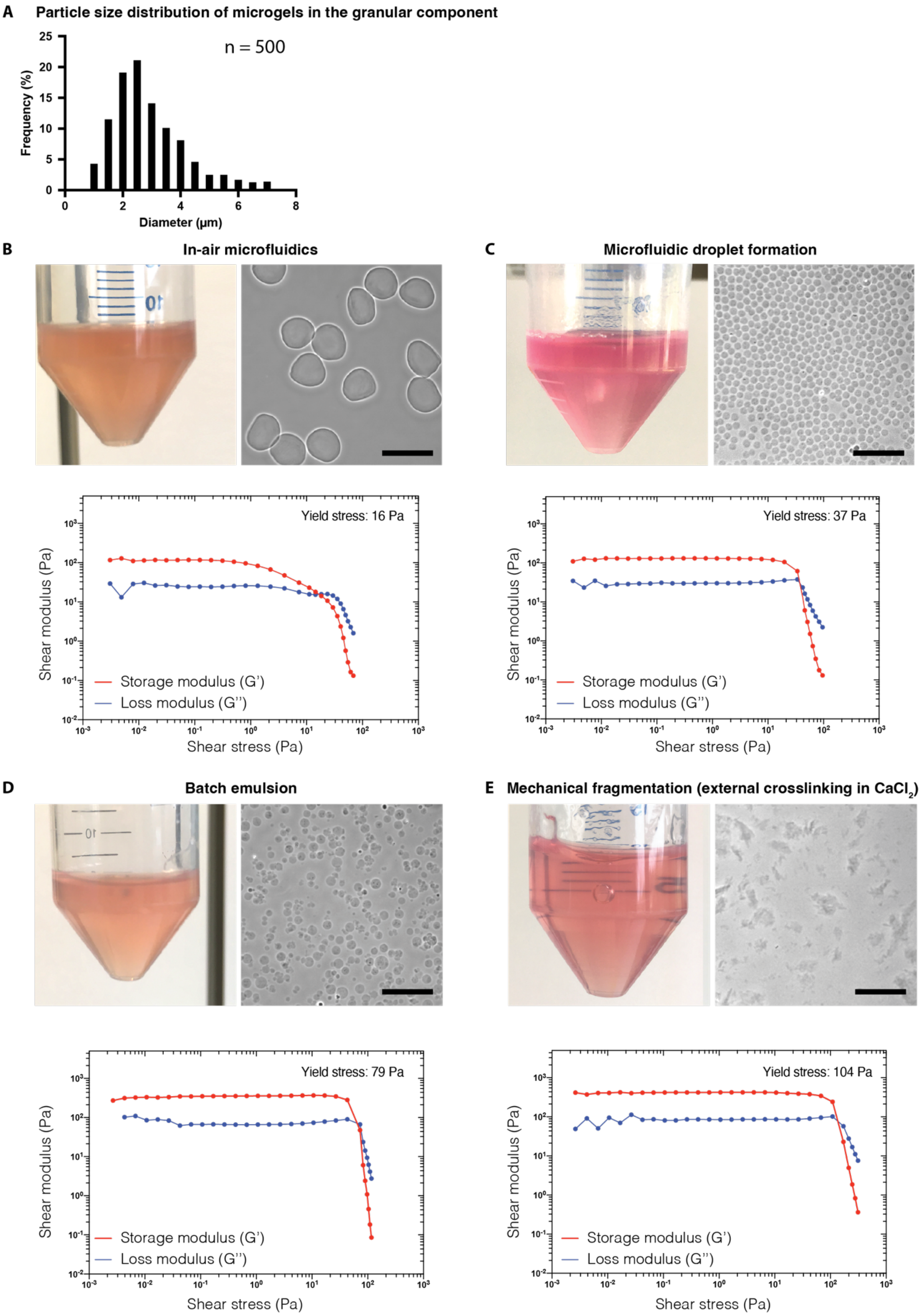
A
Particle size distribution of microgels in the granular component
n = 500
Frequency (%)
Diameter (µm)
B
In-air microfluidics
C
Microfluidic droplet formation
Yield stress: 16 Pa
Yield stress: 37 Pa
Shear modulus (Pa)
Storage modulus (G')
Loss modulus (G'')
Shear stress (Pa)
D
Batch emulsion
E
Mechanical fragmentation (external crosslinking in CaCl2)
Yield stress: 79 Pa
Yield stress: 104 Pa

Fig. S2. Particle size distribution and alternative approaches for alginate microgel fabrication. (**A**) Alginate microparticles used in SHAPE composite support generated via shearing during internal gelation followed by homogenization were imaged using light microscopy and their diameter measured. Furthermore, four different ways of generating alginate microparticles is presented in each panel with a photograph of particle slurry in the jam-packed state (showing transparency of the material), phase contrast microscopy image (showing particle shape and size), and measurement (n = 3) of shear moduli as a function of applied strain amplitude (showing that each jam-packed slurry undergoes shear stress-induced solid-to-liquid transition). (**B**) Microparticles generated using in-air microfluidics (supplied by IamFluidics, The Netherlands). (**C**) Microgels generated using flow focusing method in a polydimethylsiloxane (PDMS) microfluidic device designed and fabricated in-house where alginate solution (0.5 mg/ml, dispersed phase phase) with 1 mg mL^{-1} $CaCO_3$ was injected perpendicular to two streams of hexadecane (oil, continuous phase). The instabilities caused by surface tension led to droplet formation. Delayed addition of acetic acid (1:500 in hexadecane) to the flow after droplet formation ensures robust crosslinking of alginate hydrogels. The approach provides microgels with low polydispersity (~18 µm in diameter) but at low throughput. (**D**) Homogenization of alginate-in-oil emulsion allows higher throughput but without stringent control over microgel size. Alginate solution (0.5 mg mL^{-1} with 1 mg mL^{-1} $CaCo_3$) and hexadecane (1:3 ratio) were homogenized at 6000 rpm for 5 min. Then, same volume of. hexadecane with 0.4% acetic acid was added to the emulsion to initiate alginate crosslinking. (**E**) Mechanical fragmentation of externally crosslinked alginate hydrogels leads to flake-like microparticles with high polydispersity that, unlike the other three methods, results in highly transparent material when jam-packed. To generate these microparticles, 0.5% alginate solution was loaded into a syringe and dripped through a 500 µm ID needle into a 1% $CaCl_2$ bath thus creating a large amount of ~1 mm alginate beads that were then fragmented in a tabletop blender for 60 s. Scale bar 100 µm.

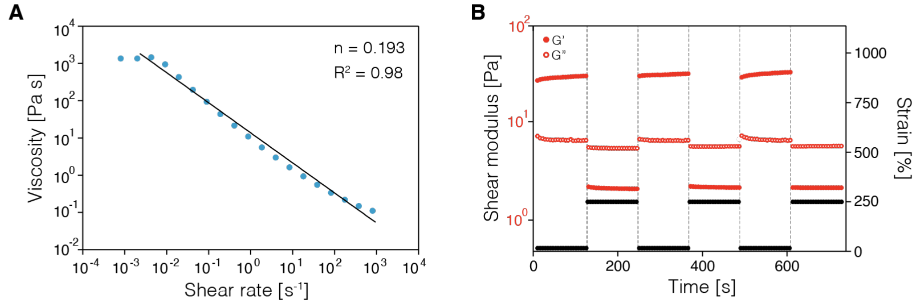

Fig. S3. Further rheological analysis of SHAPE composite support material. (A) Shear rate sweeps (n = 3) with plotted points representing the average value indicate the shear thinning property of the material. The solid line presents fit to the power-law model in the shear thinning region with power-law index n indicated in the graph. **(B)** Thixotropic test demonstrating that SHAPE support can undergo several cycles of reversible solid-to-liquid transition. Shear recovery of the material was investigated by applying alternating periods (2 min each) of 1% and 250% oscillatory strain and measuring the corresponding shear moduli.

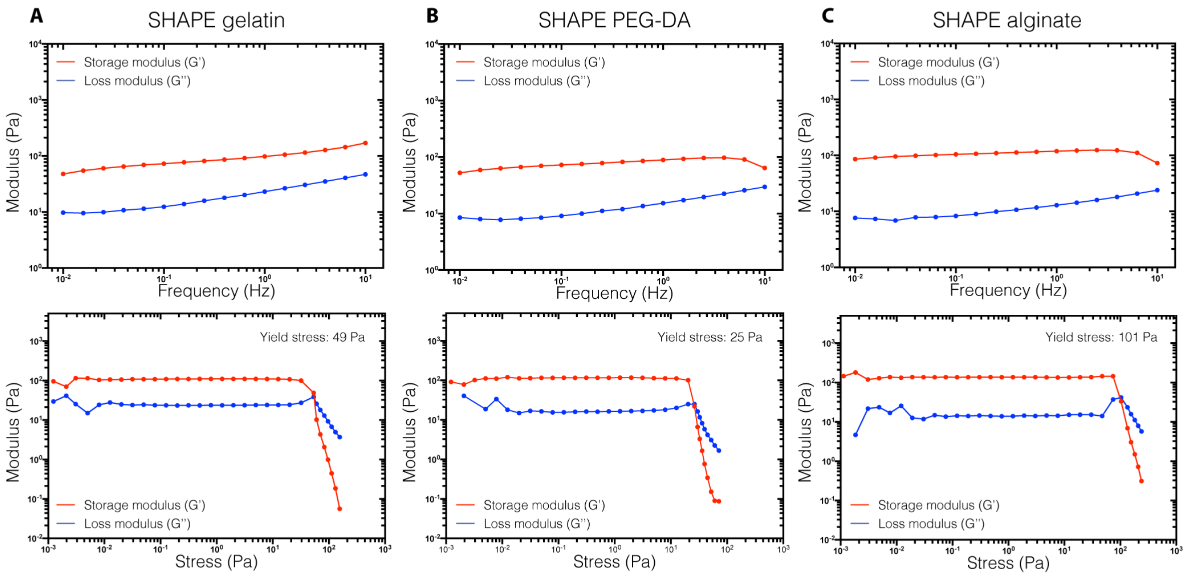

Fig. S4. Rheological characterization of alternative SHAPE hydrogel formulations. While keeping the same granular component (mechanically fragmented microgels from externally crosslinked alginate), we varied the contents of the continuous phase in order to showcase the versatility of the material landscape that can be explored for the generation of SHAPE hydrogels with different crosslinking mechanisms and for different biomanufacturing applications. In each column, the frequency sweep displayed in the top row shows that storage (G') and loss moduli (G") of alternative SHAPE hydrogel formulations are flat and show weak frequency dependence. Measurements of shear moduli as a function of applied strain amplitude (middle row) show solid-to-liquid transition. (**A**) Gelatin (10% w/v, bovine skin, Sigma Aldrich) was crosslinked enzymatically using microbial transglutaminase (Activa TI Transglutaminase, 10 U mL^{-1} in PBS) The crosslinking was performed overnight at 37 °C after which the crosslinked hydrogel was removed from the mold and enzyme washed away. (**B**) Poly(ethylene glycol) diacrylate (PEG-DA, 700 g mol^{-1}, 30% w/v, Sigma Aldrich) was crosslinked in the presence of photoinitiator (LAP, 0.5% w/v, Sigma Aldrich) using UV light with 365 nm wavelength in a curing chamber (CUREbox CB-4230, Wicked Engineering). After curing, the annealed construct was removed from the mold. (**C**) Alginate (1% w/v) was ionically crosslinked by adding 0.5% w/v $CaCl_2$ solution on top of the composite material and incubating overnight to allow Ca^{2+} diffusion throughout the printing support. Crosslinking of alginate lead to ~50% shrinkage of the support.

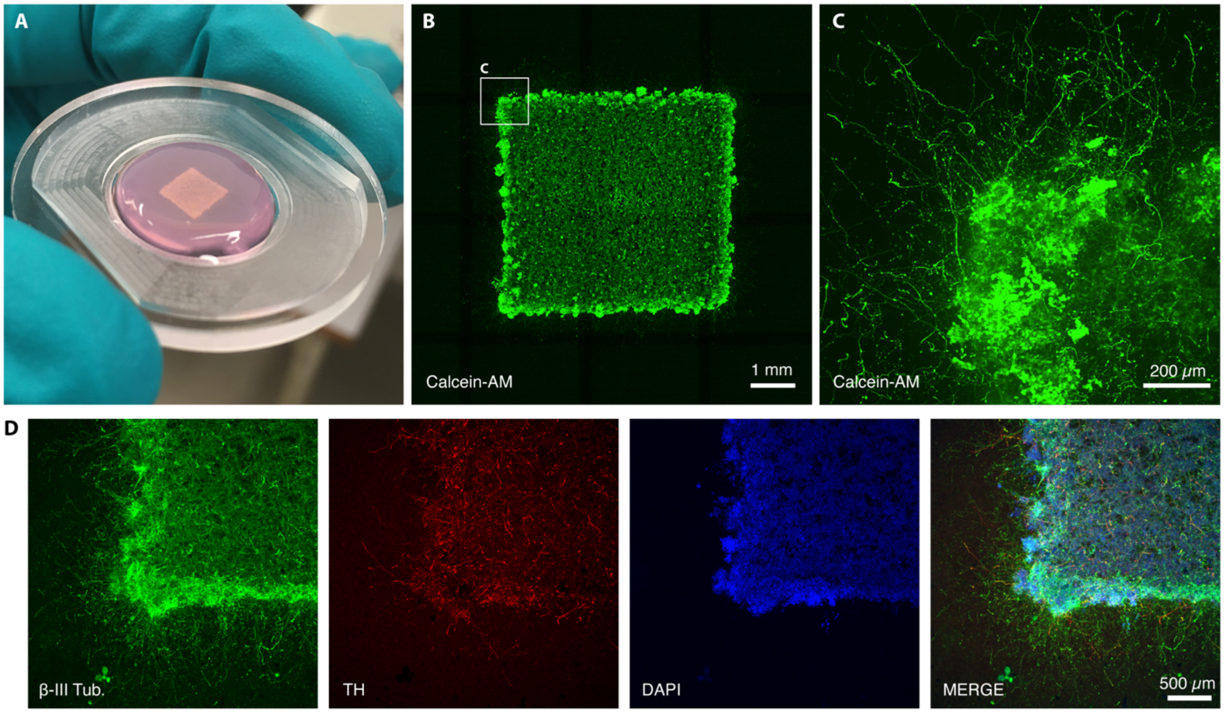

Fig. S5. Generation of human dopaminergic neurons within 3D printed construct with dense infill. (**A**) Photograph of a 3D printed square construct in annealed SHAPE hydrogel support after 2 months of differentiation. (**B**) Confocal image of the same construct labelled with live stain (Calcein-AM). (**C**) Maximum intensity projection (200 µm optical section) of a higher magnification fluorescence confocal image showing that neural projections extend away from the somatic volume and into the bulk of the support. (**D**) Fluorescence images of immunostained 3D printed construct after 2 months of differentiation displaying neuronal and dopaminergic markers (β-III tubulin and TH respectively).

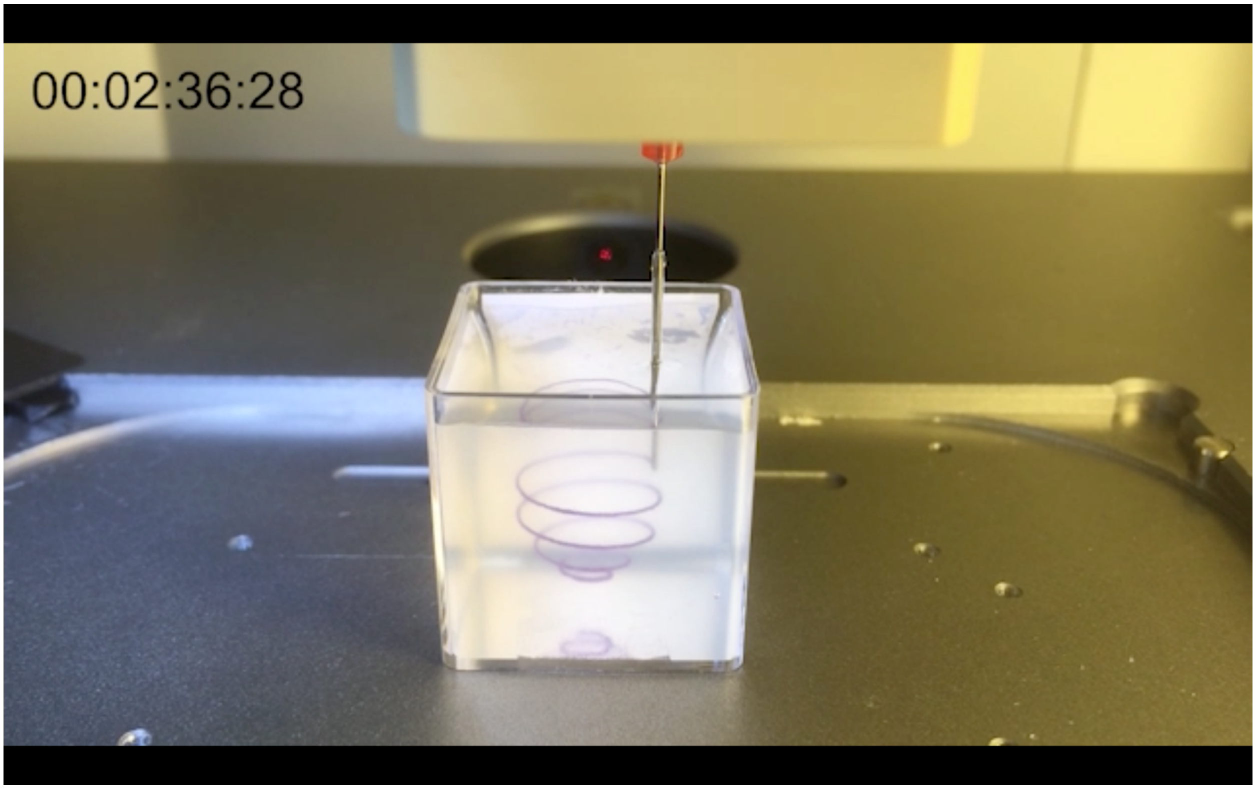

Movie S1. Omnidirectional printing in SHAPE hydrogel support. The movie shows the printing process of a spherical spiral using polystyrene beads as an ink to visualize the structure. Speed in the movie is increased to 11x. The width of the container is 25 mm.

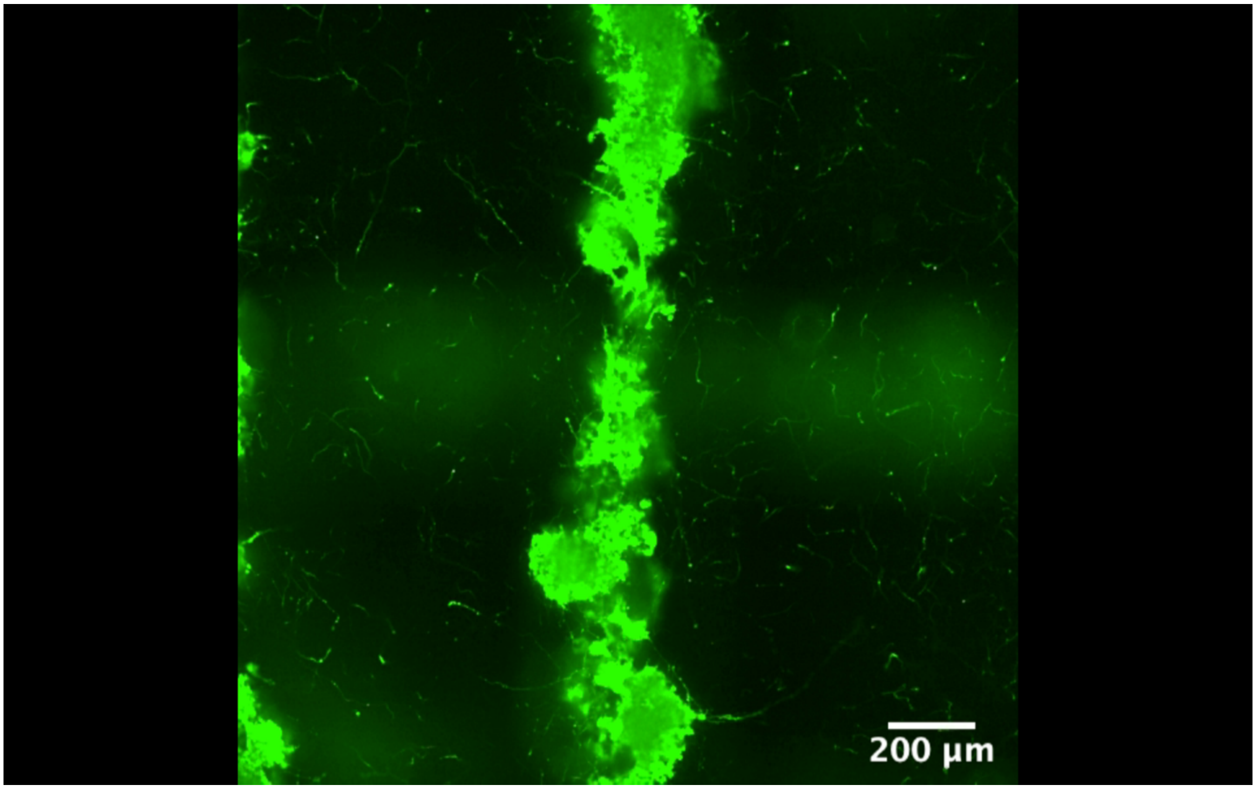

Movie S2. Neuronal projections extend throughout the 3D space of SHAPE hydrogel. The movie shows a walk-through along the z-axis for a stack of images (200 µm optical section) taken using a confocal microscope of neurons labelled with live stain (Calcein-AM) 2 months after the structure was printed and hNSC differentiation started.

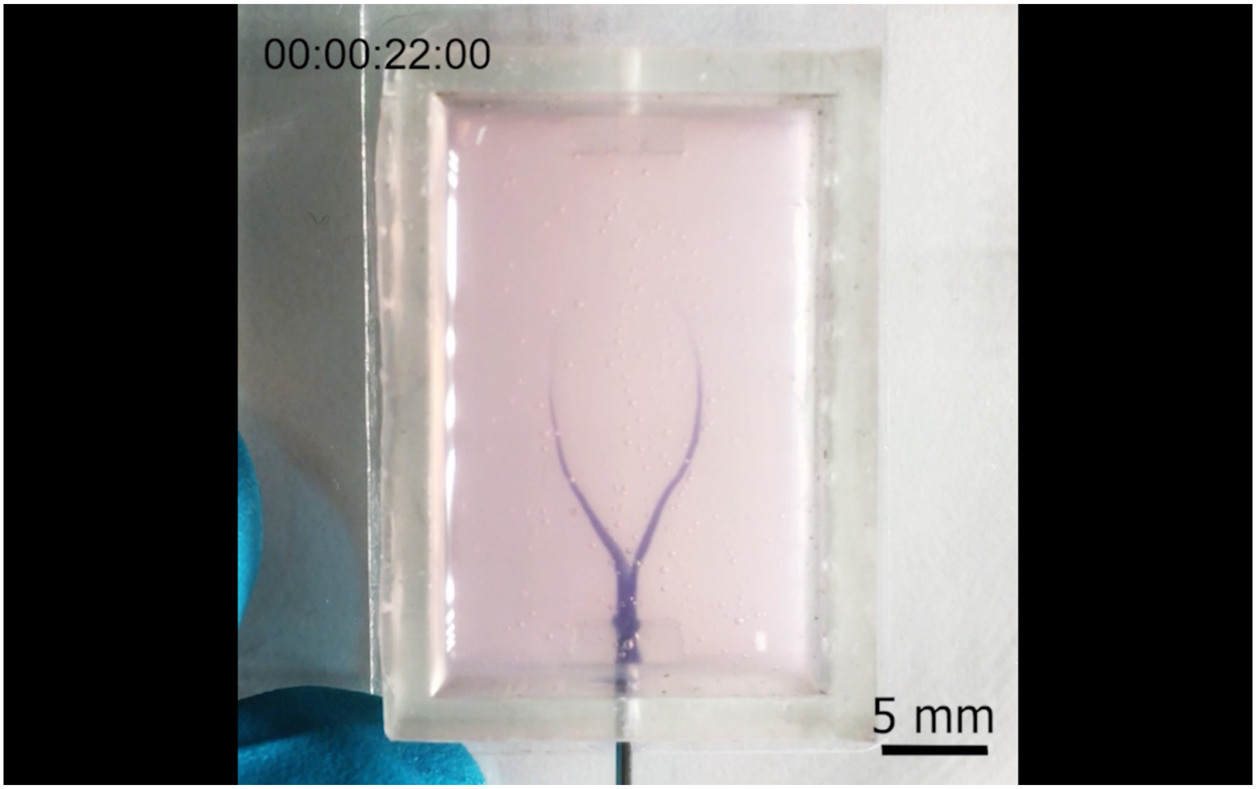

Movie S3. Sacrificial ink displacement in a branching channel. The movie shows displacement of a sacrificial gelatin ink from an annealed SHAPE hydrogel support. The material in channels is displaced simultaneously indicating successful strand fusion at the branching points during printing. Speed in the movie is increased to 6x.
